# Piceatannol suppresses the metastatic potential of MCF10A human breast epithelial cells harboring mutated H-ras by inhibiting MMP-2 expression

**DOI:** 10.3892/ijmm.2013.1449

**Published:** 2013-07-18

**Authors:** NURY SONG, MUN KYUNG HWANG, YONG-SEOK HEO, KI WON LEE, HYONG JOO LEE

**Affiliations:** 1 WCU Biomodulation Major, Department of Agricultural Biotechnology and Center for Food and Bioconvergence, Seoul National University, Seoul 151-921, Republic of Korea; 2 Advanced Institutes of Convergence Technology, Seoul National University, Suwon 443-270, Republic of Korea; 3 Department of Chemistry, Konkuk University, Seoul 143-701, Republic of Korea

**Keywords:** H-ras, matrix metalloproteinase-2, metastasis, phosphatidylinositol 3-kinase, piceatannol

## Abstract

Metastasis is one of the most threatening features of the oncogenic process and the main cause of cancer-related mortality. Several studies have demonstrated that matrix metalloproteinases (MMPs) are critical for tumor invasion and metastasis. Resveratrol (3,5,4′-trihydroxystilbene), a phenolic compound of red wine, has been reported to be a natural chemopreventive agent. However, the cancer preventive effects of piceatannol (3,5,3′,4′-tetrahydroxystilbene), a metabolite of resveratrol and the underlying molecular mechanisms have not yet been fully elucidated. In this study, we report that piceatannol inhibits H-ras-induced MMP-2 activity and the invasive phenotype of MCF10A human breast epithelial cells harboring mutated H-ras (H-ras MCF10A cells) more effectively than resveratrol. Piceatannol attenuated the H-ras-induced phosphorylation of Akt in a time- and dose-dependent manner, whereas resveratrol, at the same concentrations, did not exert an inhibitory effect. *In vitro* kinase assays demonstrated that piceatannol significantly inhibited phosphatidylinositol 3-kinase (PI3K) activity and suppressed phosphatidylinositol (3,4,5)-trisphosphate (PIP_3_) expression in the H-ras MCF10A cells. *Ex vivo* pull-down assays revealed that piceatannol directly bound to PI3K, inhibiting PI3K activity. Data from molecular docking suggested that piceatannol is a more tight-binding inhibitor than resveratrol due to the additional hydrogen bond between the hydroxyl group and the backbone amide group of Val882 in the ATP-binding pocket of PI3K.

## Introduction

Tumor invasion and metastasis are among the most threatening features of the oncogenic process and are the cause of 90% of human cancer-related deaths (1). Tumor cell invasion and metastasis are complex processes involving extracellular matrix (ECM) degradation and cell migration through the ECM (2,3). Matrix metalloproteinases (MMPs) are a family of zinc-dependent endopeptidases that play a crucial role in invasion and metastasis through the degradation of the ECM (4). MMPs are upregulated in the majority of human cancers and uncontrolled proteolysis plays a crucial role in several pathological conditions (5). Several studies have demonstrated that MMP-2 and MMP-9 are critical for tumor invasion and metastasis by degrading collagen IV, a main component of basement membranes (6–8). A previous study indicated that MMP-2, as opposed to MMP-9, is closely associated with invasion and migration in MCF10A human breast epithelial cells harboring mutated H-ras (H-ras MCF10A cells) (9).

The uncontrolled activation of the *ras* signaling pathway is one of the most frequent defects in human cancers (10). In human breast cancer, *ras* mutations are infrequent, but upregulated amounts of the ras protein have been found in 60–70% of human breast cancers (11). A number of studies have suggested that the expression of ras is associated with the invasiveness of breast cancer cells and is crucial for tumor aggressiveness, including tumor recurrence, degree of invasion of fat tissues and infiltration into lymphatic vessels (11–13). A previous study indicated that H-ras, but not N-ras, induces the invasive phenotype of MCF10A cells and suggested the important role of MMP-2 in cell invasion (14).

The best characterized effectors of Ras are the Raf kinases (15) and phosphatidylinositol 3-kinases (PI3Ks) (16). PI3Ks are heterodimeric lipid kinases that are involved in various cellular responses, including cell growth, proliferation, cytoskeletal rearrangement and motility (17). Activated PI3K synthesizes the second messenger, phosphatidylinositol (3,4,5)-trisphosphate (PIP_3_), from phosphatidylinositol (4,5)-bisphosphate (PIP_2_), thereby affecting the activation of phosphoinositide-dependent kinases (PDKs) and Akt signaling (18). The accumulation of PIP_3_ creates a docking site for Akt at the plasma membrane, which binds to PIP_3_ via the pleckstrin homology domain. Akt is a serine/threonine kinase and its activation has been implicated in the genesis or progression of various human malignancies (17,19). Significantly upregulated PI3K activity has been detected in highly invasive MDA-MB-231 cells (20). One of the characteristics of highly invasive cancers is the aberrant activation of nuclear factor-κB (NF-κB) and PI3K activates NF-κB through distinct mechanisms in different cell lines (20,21). It has previously been demonstrated that the PI3K/Akt signaling pathway is involved in the invasive phenotype of H-ras MCF10A cells (9).

**Figure 1:**
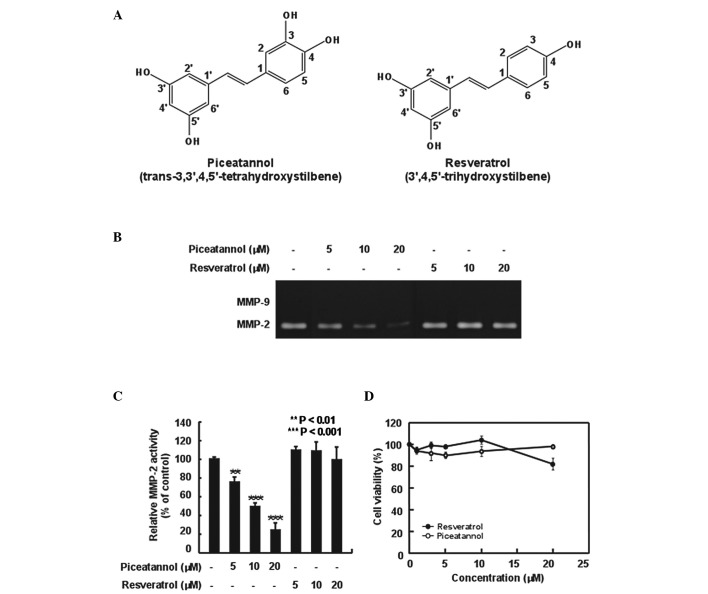
Effects of piceatannol and resveratrol on matrix metalloproteinase-2 (MMP-2) activity in H-ras MCF10A cells. (A) Chemical structures of piceatannol and resveratrol. (B) Effect of piceatannol and resveratrol on MMP-2 activity in H-ras MCF10A cells. Cells were starved in serum-free DMEM/F12 for 24 h and then treated with samples at the indicated concentrations for 24 h. The conditioned medium was analyzed by zymography, as described in Materials and methods. (C) The relative level of MMP-2 activity was quantified densitometrically using Image J software. Data are representative of three independent experiments that produced similar results. (D) Effects of piceatannol and resveratrol on H-ras MCF10A cell viability. Cells were treated for 24 h in the presence or absence of piceatannol or resveratrol. Cell viability was quantified using MTT assay, as described in Materials and methods. Data are the means ± SD of three independent experiments.

Resveratrol (3,5,4′-trihydroxystilbene; Fig. 1A), a phenolic compound of red wine, has been reported to be a natural chemopreventive agent against several types of cancer, such as leukemia (22), breast (23) and prostate cancer (24). Piceatannol (3,5,3′,4′-tetrahydroxystilbene; Fig. 1A) is a naturally occurring polyphenol and a metabolite of the cancer chemopreventive agent, resveratrol, via cytochrome P4501B1, which is overexpressed in a variety of human tumors (25). It has been demonstrated that piceatannol exerts anti-inflammatory (26) and anticancer effects (27). However, the cancer preventive effects of piceatannol and the underlying molecular mechanisms have not yet been fully elucidated. In this study, we investigated the possible inhibitory effects of piceatannol on the H-ras-induced invasiveness of MCF10A human breast epithelial cells, as well as the underlying mechanisms.

## Materials and methods

### Chemicals

Piceatannol was obtained from A.G. Scientific, Inc. (San Diego, CA, USA). Resveratrol was purchased from Sigma-Aldrich (St. Louis, MO, USA). LY294002 and triciribine were obtained from Calbiochem (San Diego, CA, USA). Dulbecco’s modified Eagle’s medium (DMEM)/F12, horse serum, L-glutamine and the penicillin/streptomycin/fungizone mixture were purchased from Gibco-BRL (Grand Island, NY, USA). Protein A/G sepharose beads were purchased from Santa Cruz Biotechnology, Inc. (Santa Cruz, CA, USA). The antibodies against phosphorylated Akt (Ser473) and total Akt were obtained from Cell Signaling Technology (Beverly, MA, USA). The antibodies against PI3K p85 and p110 were obtained from Upstate Biotechnology (Lake Placid, NY, USA). CNBr-Sepharose 4B and [γ-^32^P]ATP were purchased from Amersham Pharmacia Biotech (Piscataway, NJ, USA) and the protein assay kit was from Bio-Rad Laboratories (Hercules, CA, USA).

### Cell culture

H-ras MCF10A cells (kindly supplied by Dr Aree Moon, Duksung Women’s University, Seoul, Korea) were cultured in DMEM/F12 supplemented with 5% heat-inactivated horse serum, 10 μg/ml insulin, 100 ng/ml cholera toxin, 0.5 μg/ml hydrocortisone, 20 ng/ml recombinant epidermal growth factor, 2 mM L-glutamine and 100 ng/ml penicillin/streptomycin/fungizone mixture in a 37°C humidified atmosphere of 5% CO_2_/95% air.

### Gelatin zymography for MMPs

The H-ras MCF10A cells (5×10^5^) plated on culture dishes at 90% confluence were maintained in serum-free medium for 24 h. The cells were cultured in serum-free medium containing chemicals or chemical inhibitors for a further 24 h. Conditioned medium was collected and concentrated at 10,000 × g for 1 h in a SpeedVac concentrator (Savant/E-C Instruments, Niantic, CT, USA). The protein concentration was measured using a dye-binding protein assay kit (Bio-Rad Laboratories) as described in the manufacturer’s manual. Equal amounts of protein from the conditioned medium were mixed with 2X non-reducing sample buffer, incubated for 5 min at room temperature and then electrophoresed on 10% sodium dodecyl sulfate (SDS)-polyacrylamide gel electrophoresis (PAGE) gels containing 1 mg/ml gelatin. After electrophoresis, the gels were washed with 2.5% Triton X-100 three times each for 30 min to remove SDS, rinsed two times each for 10 min with a 50 mM Tris-HCl buffer (pH 7.6) containing 5 mM CaCl_2_, 200 mM NaCl and 0.02% Brij-35 and then incubated overnight at 37°C. The gels were stained with 0.5% Coomassie brilliant blue R-250 solution containing 10% acetic acid and 20% methanol for 30 min and then destained with 7.5% acetic acid solution containing 10% methanol. Areas of gelatinase activity appear as clear bands (zones of gelatin degradation) against the blue-stained gelatin background.

### Cell viability assay

To estimate cell viability, H-ras MCF10A cells were seeded in 96-well plates (n=3) and grown to near confluence in conditioned medium. The H-ras MCF10A cells were then starved for 24 h. The cells were either treated or not treated with various concentrations of piceatannol or resveratrol (1.25–20 μM) for 24 h. Subsequently, 20 μl of MTT solution (0.5 mg/ml) were added to each well containing 200 μl of conditioned medium and incubated for a further 4 h at 37°C. The medium was then removed and 200 μl of dimethyl sulphoxide (DMSO) were added to each well. After shaking, cell viability was determined by reading the absorbance at 570 nm and the results were expressed as the cell viability ratio relative to the untreated control.

### Invasion assay

An invasion assay was performed using the Transwell system (Corning Costar Co., Cambridge, MA, USA). The lower side of the filter was coated with 10 μl of type I collagen and the upper side was coated with Matrigel (Upstate Biotechnology). The lower compartment was filled with 600 μl of serum-free medium containing 0.1% bovine serum albumin (BSA). The H-ras MCF10A cells (5×10^4^) were resuspended with samples in 100 μl of medium and placed in the upper part of the Transwell plate. The cells were then incubated for 24 h in a humidified atmosphere of 5% CO_2_ at 37°C. The cells on the upper surface of the filter were completely wiped off with a cotton swab, fixed with methanol and stained with hematoxylin for 10 min. After washing in water, the cells were stained with eosin for 4 min. The invading cells were quantified by counting the cells that had migrated to the lower side of the filter under a microscope. Thirteen randomly selected fields were counted and each sample was assayed in duplicate.

### Wound migration assay

The H-ras MCF10A cells (5×10^5^) were plated on culture dishes and grown to 90% confluence in 2 ml of growth medium. The cells were cultured with mitomycin C (25 μg/ml) for 30 min and then an injury line was made using a plastic pipette tip (all tips used had the same width). The plates were rinsed with phosphate-buffered saline (PBS) and then complete medium containing the samples was added. The cells were allowed to migrate in the medium and images were acquired using an inverted microscope (x100/x200 magnification; Olympus Ix70, Okaya, Japan) at specific time points. The width of the injury line was then measured in three independent experiments and was plotted as a percentage of the width at 0 h.

### Western blot analysis

After the cells were cultured for 24 h, they were starved in serum-free DMEM/F12 for a further 24 h. The cells were then treated with chemicals for 30 min. Cell lysates were scraped and treated with lysis buffer [10 mM Tris (pH 7.5), 150 mM NaCl, 5 mM EDTA, 1% Triton X-100, 1 mM DTT, 0.1 mM PMSF, 10% glycerol and a protease inhibitor cocktail tablet] for 40 min on ice followed by centrifugation at 14,000 rpm for 10 min. The protein concentration of the supernatant was determined using a dye-binding protein assay kit (Bio-Rad Laboratories) as described in the manufacturer’s manual. Lysate protein (30 μg) was subjected to 10% SDS-PAGE and electrophoretically transferred onto a polyvinylidene difluoride (PVDF) membrane (Immobilon P, Millipore Corp., Bedford, MA, USA). After blotting, the membrane was blocked in 5% fat-free dry milk for 1 h and then incubated with the specific primary antibody for 2 h at room temperature. Protein bands were detected by using an enhanced chemiluminescence (ECL) detection kit (Amersham) after hybridization with the HRP-conjugated secondary antibody.

### In vitro PI3K assay

An active PI3K protein (80 ng) was incubated with piceatannol or LY294002 at the indicated concentrations for 10 min at 30°C. The mixtures were then incubated with 20 μl of 0.5 mg/ml phosphatidylinositol (Avanti Polar Lipids, Inc., Alabaster, AL, USA). After 5 min at room temperature, the mixtures were incubated with reaction buffer [100 mM HEPES (pH 7.6), 50 mM MgCl_2_, 250 μM ATP containing 10 μCi of [γ-^32^P]ATP] for an additional 10 min at 30°C. The reaction was terminated by the addition of 15 μl of 4 N HCl and 130 μl of chloroform/methanol (1:1). After vortexing, 30 μl of the lower chloroform phase was spotted onto a 1% potassium oxalate-coated silica gel plate, which was previously activated for 1 h at 110°C. The resulting ^32^P-labeled phosphatidylinositol-3-phosphate (PIP) was separated by thin layer chromatography (TLC) and radiolabeled spots were visualized by autoradiography.

### Immunofluorescence assay

Immunofluorescent staining was conducted on the H-ras MCF10A cells cultured on cover slips. All staining procedures were performed on ice or at 4°C unless otherwise stated. The cells were grown on coverslips and cultured for 24 h. Following treatment with the indicated doses of piceatannol for 15 min, the cells were fixed with 4% formaldehyde for 15 min at room temperature and permeabilized with ice-cold 100% methanol for 10 min. The cells were stained with PIP_3_ primary antibody followed by FITC-conjugated secondary antibody at dilutions recommended by the manufacturer and cell nuclei were stained with DAPI.

### Ex vivo pull-down assay

An H-ras MCF10A cellular supernatant fraction (500 μg) was incubated with piceatannol-Sepharose 4B (or Sepharose 4B as the control) beads (100 μl, 50% slurry) in reaction buffer [50 mM Tris-HCl (pH 7.5), 5 mM EDTA, 150 mM NaCl, 1 mM DTT, 0.01% Nonidet P-40, 2 μg/ml bovine serum albumin, 0.02 mM PMSF and 1X protease inhibitor mixture]. Following incubation with gentle rocking overnight at 4°C, the beads were washed five times with buffer [50 mM Tris-HCl (pH 7.5), 5 mM EDTA, 150 mM NaCl, 1 mM DTT, 0.01% Nonidet P-40 and 0.02 mM PMSF] and proteins bound to the beads were analyzed by immunoblotting.

### Molecular modeling

Insight II (Accelrys, Inc., San Diego, CA, USA) was used for molecular docking and structure analysis; the crystal coordinates of PI3K in the complex with ATP (accession code 1E8X) are available on the Protein Data Bank (http://www.rcsb.org/pdb/).

### Statistical analysis

Where appropriate, data are expressed as the means ± SD and the Student’s t-test was used to perform statistical analysis for single comparison. Probability values of p<0.05 were considered to indicate statistically significant differences.

## Results

### Piceatannol inhibits H-ras-induced MMP-2 activity more effectively than resveratrol in MCF10A cells

MMP-2 and MMP-9 are critical enzymes for ECM degradation, which is a critical step in tumor metastasis. A previous study demonstrated that the H-ras-induced invasion of MCF10A cells is associated more closely with the expression of MMP-2 than of MMP-9 (9). In this study, to investigate the protective effect of piceatannol against cancer metastasis, we first examined the inhibitory effect of piceatannol on MMP-2 upregulation in H-ras MCF10A cells. Piceatannol, but not resveratrol, inhibited the upregulation of MMP-2 activity in the H-ras MCF10A cells in a dose-dependent manner (Fig. 1B and C). The survival of the cells treated with piceatannol for up to 24 h was comparable to that of the cells at 0 h, as determined by MTT assay (Fig. 1D), indicating that the inhibition of MMP-2 upregulation was not due to piceatannol cytotoxicity.

### Piceatannol suppresses H-ras-induced cell invasion and migration more effectively than resveratrol in MCF10A cells

**Figure 2:**
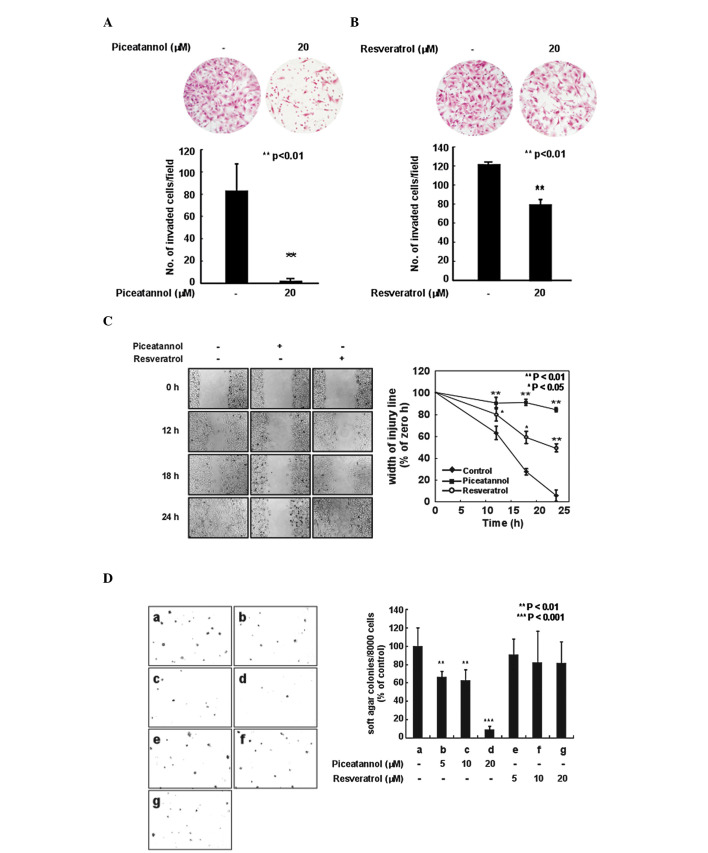
Inhibitory effects of piceatannol and resveratrol on the invasion, migration and neoplastic transformation of H-ras MCF10A cells. (A and B) Invaded cells were quantified by counting the cells that migrated to the lower side of the filter in random microscopic fields. Each bar indicates the mean ± SD (n=3). ^*^p<0.05, ^**^p<0.01 compared with the control. (C) Confluent H-ras MCF10A cells in serum-free medium were treated with resveratrol or piceatannol. Thereafter, the widths of the injury lines were measured at 0, 12, 18 and 24 h. Results are expressed as the widths of the injury lines relative to the untreated controls at 0 h, as determined from three independent experiments. Data are the means ± SD. (D) Comparison of the inhibitory effects of piceatannol and resveratrol on the H-ras-induced neoplastic transformation of MCF10A cells. Cells were treated as described in Materials and methods and the number of colonies was determined 15 days later: (a) untreated control; (b) piceatannol 5 μM; (c) piceatannol 10 μM; (d) piceatannol 20 μM; (e), resveratrol 5 μM; (f) resveratrol 10 μM; (g) resveratrol 20 μM. The colonies were counted under a microscope using Image-Pro Plus software version 4. Data are presented as the mean numbers of colonies ± SD, as determined by three independent experiments. ^**^p<0.01 and ^***^p<0.001, significant difference between the group treated with piceatannol or resveratrol and the untreated group.

Invasion and migration are important phenotypes in cancer metastasis (28). In this study, we examined the effects of piceatannol and resveratrol on cell invasion and migration in H-ras MCF10A cells. The H-ras-induced invasion of MCF10A cells was markedly inhibited following treatment with piceatannol at a concentration of 20 μM (Fig. 2A). Resveratrol also inhibited the invasive ability of the H-ras MCF10A cells (Fig. 2B), although piceatannol suppressed the H-ras-induced invasive ability of the cells more effectively than did resveratrol. Subsequently, we investigated the effects of piceatannol and resveratrol on the H-ras-induced cell migration of MCF10A cells. As expected, piceatannol inhibited the cell migration ability at a concentration of 20 μM (Fig. 2C). Resveratrol also suppressed the migration ability of the H-ras MCF10A cells; however, its effects were not as prominent as those of piceatannol. Treatment with piceatannol significantly reduced the H-ras-induced anchorage-independent growth of MCF10A cells in a dose-dependent manner (Fig. 2D). Based on the number of colonies, piceatannol at 20 μM suppressed the H-ras-induced anchorage-independent growth by 90%. However, resveratrol did not significantly inhibit H-ras-induced anchorage-independent growth.

### Piceatannol attenuates the H-ras-induced phosphorylation of Akt and PI3K activity in MCF10A cells

**Figure 3:**
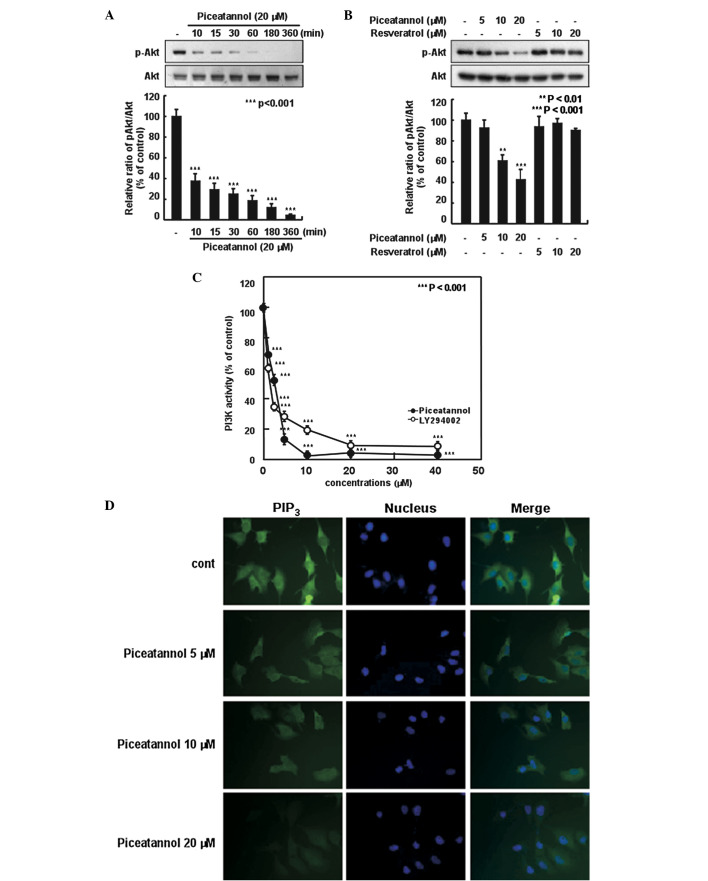
Effects of piceatannol and resveratrol on the phosphorylation of Akt and phosphatidylinositol 3-kinase (PI3K) activity in H-ras MCF10A cells. (A) After 24 h of serum starvation, the cells were treated with 20 μM piceatannol for the indicated periods of time. (B) After 24 h of starvation, the cells were treated with 5, 10, or 20 μM piceatannol or resveratrol for 15 min. The levels of phosphorylated and total Akt protein were determined by western blot analysis, as described in Materials and methods, using specific antibodies against the corresponding phosphorylated and total protein. Each experiment was performed in triplicate. (C) Effects of piceatannol and LY294002 on PI3K activity *in vitro*. Active PI3K (80 ng) was pre-incubated with piceatannol and LY294002 at 1.25, 2.5, 5, 10, 20, or 40 μM for 10 min at 30°C, then incubated with phosphatidylinositol substrate and [γ-^32^P]ATP for an additional 10 min at 30°C. The resulting ^32^P-labeled phosphatidylinositol phosphate (PIP) was measured as described in Materials and methods. The relative level of PI3K activity was quantified densitometrically using Image J software. Data are the means ± SD of three independent experiments. (D) An immunofluorescence assay was performed on the cells for PI3K activity using anti-PIP_3_ antibody. The immunostained cells (green) were observed under a confocal microscope.

To elucidate the inhibitory mechanisms of piceatannol on MMP-2 activity, we investigated the effects of piceatannol on the phosphorylation of Akt and PI3K activity in the H-ras MCF10A cells. Piceatannol markedly suppressed the H-ras-induced phosphorylation of Akt in a time- and dose-dependent manner in the MCF10A cells (Fig. 3A and B). We also found that 20 μM of piceatannol almost completely inhibited Akt phosphorylation in the H-ras MCF10A cells; however, the same concentration of resveratrol did not exert an inhibitory effect (Fig. 3B). We also investigated whether piceatannol affects the levels of p38 and extracellular signal-regulated kinase (ERK) phosphorylation and found that it did not inhibit p38 and ERK phosphorylation (data not shown). These results suggest that piceatannol regulates Akt phosphorylation without affecting p38 and ERK phosphorylation. Moreover, in order to determine the direct target of piceatannol, we investigated the effects of piceatannol on PI3K activity, the upstream kinase of Akt, *in vitro*. Piceatannol markedly suppressed PI3K activity and its effects were similar to those of LY294002, a commercial PI3K inhibitor (Fig. 3C). Activated PI3K generates PIP_3_ and phosphorylates Akt (17). Therefore, we then examined the inhibitory effects of piceatannol on PI3K activity in the H-ras MCF10A cells by the immunostaining of PIP_3_. Piceatannol inhibited PIP_3_ expression in a dose-dependent manner in the H-ras MCF10A cells (Fig. 3D). These results suggest that piceatannol exerts its antimetastatic effects through the direct inhibition of PI3K activity.

### PI3K pathway is involved in H-ras-induced MMP-2 activity, invasion and migration in MCF10A cells

**Figure 4:**
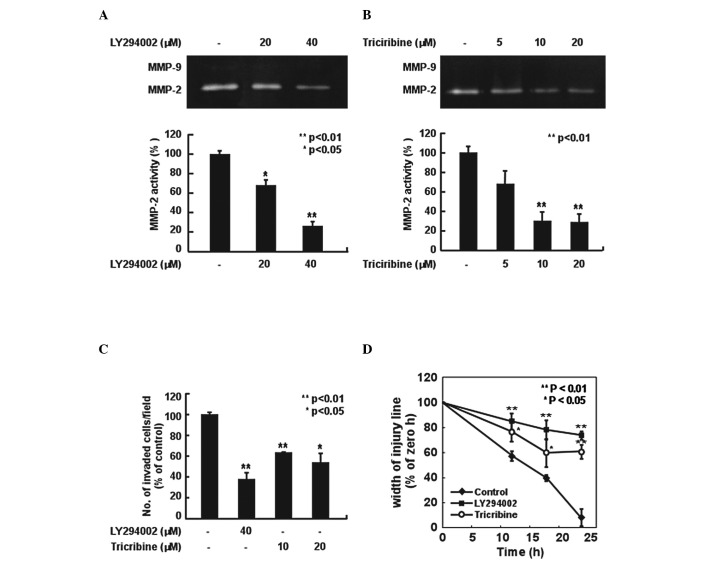
Effects of the phosphatidylinositol 3-kinase PI3K inhibitor (LY294002) and Akt inhibitor (triciribine) on H-ras-induced matrix metalloproteinase-2 (MMP-2) activity, and on the migration and invasion of MCF10A cells. (A and B) Cells were starved in serum-free DMEM/F12 for 24 h and then treated with samples at the indicated concentrations for 24 h. The conditioned medium was analyzed by zymography, as described in Materials and methods. (C) Invaded cells were quantified by counting the cells that had migrated to the lower side of the filter in random microscopic fields. Each bar indicates the mean ± SD (n=3). ^*^p<0.05, ^**^p<0.01 compared to the control. (D) The confluent H-ras MCF10A cells in serum-free medium were treated with 40 μM LY294002 or 20 μM triciribine. Thereafter, the widths of the injury lines were measured at 0, 12, 18 and 24 h. Results are expressed as the widths of the injury lines relative to untreated controls at 0 h, as determined from three independent experiments. Data are the means ± SD.

We found that piceatannol suppressed H-ras-induced Akt phosphorylation through the direct inhibition of PI3K activity. We then investigated the role of the PI3K/Akt pathway in MMP-2 expression, invasion and migration in H-ras MCF10A cells using a specific inhibitor of PI3K, LY294002, and a commercial inhibitor of Akt, triciribine. LY294002 and triciribine inhibited the H-ras-induced MMP-2 activity in the MCF10A cells (Fig. 4A and B). Moreover, these two inhibitors also suppressed the H-ras-induced cell invasion and migration ability of MCF10A cells (Fig. 4C and D). These results indicate that the PI3K/Akt pathway is involved in the induction of MMP-2 activity, as well as in the invasion and migration ability of H-ras MCF10A cells.

### Piceatannol directly binds to PI3K in H-ras MCF10A cells

**Figure 5:**
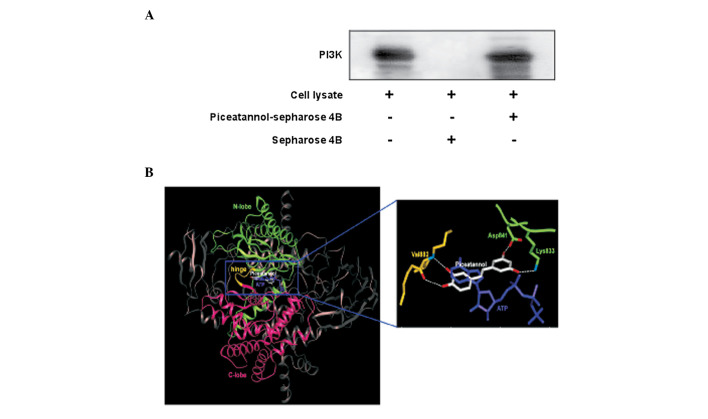
Direct binding between piceatannol and phosphatidylinositol 3-kinase (PI3K). (A) *Ex vivo* binding of PI3K and piceatannol was confirmed by immunoblotting using an antibody against PI3K: lane 1, input control using whole-cell lysate from H-ras MCF10A cells; lane 2, negative control using a precipitated lysate of H-ras MCF10A cells with Sepharose 4B beads; lane 3, whole-cell lysate from H-ras MCF10A cells precipitated by piceatannol-bound Sepharose 4B beads. (B) Hypothetical model of PI3K in complex with piceatannol. The Ras-binding domain, C2 domain and helical domain of PI3K are colored gray. The N-lobe, C-lobe and hinge region of the catalytic domain are colored green, purple and yellow, respectively. Piceatannol (atomic color) binds to the ATP binding site in the catalytic domain of PI3K. ATP (violet) is overlaid on the model structure of the complex for comparison. The hydrogen bonds are depicted as dashed lines.

To further investigate the mechanism behind the inhibitory effect of piceatannol on PI3K activity, we performed *ex vivo* pull-down assays. We detected PI3K in the piceatannol-Sepharose beads (Fig. 5A; lane 3), but not in the control-Sepharose beads (Fig. 5A; lane 2). The input lane (Fig. 5A; lane 1) indicates that only cell lysates were loaded as a marker to ensure that the band detected the PI3K protein itself. These data indicate that piceatannol inhibits PI3K activity and downstream signaling by directly binding to PI3K.

## Discussion

Several studies have demonstrated that red wine has anticarcinogenic effects (29,30). Resveratrol is a well-known active phenolic compound of red wine and has been reported to be a natural chemopreventive agent against several types of cancer (22–24). Piceatannol is a major metabolite of resveratrol and is generated by CYP1B1, a cytochrome p450 enzyme (25). A previous study indicated that resveratrol is metabolized into a glucuronidated or sulphated form within 15 min and that a moderate consumption of red wine is insufficient for resveratrol to reach an effective concentration (31). The chemical structure of piceatannol is similar to that of resveratrol, with the addition of a hydroxyl group at the 3′-position in the B-ring moiety of resveratrol (Fig. 1A). In the present study, we demonstrate that piceatannol exerts significantly greater inhibitory effects against H-ras-induced MMP-2 expression, invasion and migration than resveratrol in MCF10A cells.

Tumor metastasis, the spread of tumor cells from the primary site to distant sites, is the main cause of morbidity and mortality in cancer patients. The metastatic process of quite complex, involving the dissociation of malignant cells in the primary tumor, local invasion, angiogenesis and the intravasation of invading cells into the vasculature or lymphatic systems (32,33). It is well known that invasive tumor cells secrete matrix-degrading proteases (34) and collagen, a main component of the ECM, is an important substrate as it constitutes the structural scaffolding upon which the other components of the matrix are assembled (35). The degradation of the ECM is a crucial step in cell migration and invasion and MMPs are essential for this step (36). MMPs are a family of zinc-dependent endopeptidases and are upregulated in the majority of human cancers (5). In particular, MMP-2 and MMP-9 are collagenases and are crucial for tumor invasion and metastasis by degrading collagen IV (6–8). A previous study demonstrated that MMP-2 is more closely associated with cell invasion and migration than MMP-9 in H-ras MCF10A human breast epithelial cells (9). In this study, we demonstrate that piceatannol, but not resveratrol, inhibits H-ras-induced MMP-2 expression, significantly suppressing the migration and invasion of MCF10A cells. Moreover, we demonstrate that piceatannol suppresses the anchorage-independent growth of H-ras MCF10A cells.

Ras proteins are associated with the inner face of the plasma membrane and their activity is regulated by cycling between the inactive GDP-bound and the active GTP-bound forms (37). The upregulation of Ras activity induces all aspects of the malignant phenotype of cancer cells, including cellular proliferation, transformation, invasion and metastasis (38). Ras regulates multiple downstream effectors that stimulate diverse cytoplasmic signaling activities (39). PI3K is one of the best-characterized effectors of Ras proteins (16). This kinase phosphorylates PIP_2_ to form the second messenger, PIP_3_, which activates Akt/PKB, a serine/threonine kinase (17,19). A previous study indicated that the PI3K/Akt signaling pathway is involved in the H-ras-induced invasiveness of MCF10A human breast epithelial cells (9). In this study, we found that PI3K/Akt signaling inhibited the H-ras-induced MMP-2 expression, as well as the invasion and migration of MCF10A cells. Moreover, piceatannol exerted a greater inhibitory effect on Akt phosphorylation than resveratrol.

PI3Ks are heterodimeric lipid kinases composed of regulatory (p85) and catalytic subunits (p110) that are encoded by different genes (40,41). Previous studies have demonstrated that activated Ras protein directly binds with the p110 catalytic subunit of PI3K and further stimulates PI3K activity (42,43). In our study, we found that piceatannol markedly inhibited PI3K activity *in vitro* in the H-ras MCF10A cells. Moreover, we confirmed that piceatannol directly binds to PI3K. To investigate the molecular mechanisms behind the inhibition of PI3K by piceatannol, we carried out a modeling study using the crystal structure of PI3K in a complex with ATP or inhibitors (44,45). PI3K consists of four domains: a Ras-binding domain, a C2 domain, a helical domain and a catalytic domain. The catalytic domain of the enzyme consists of an N-lobe and a C-lobe with a fold similar to protein kinases and this structural similarity is also conserved in the ATP-binding site that is flanked by these two lobes. Consequently, ATP binds between these lobes in a manner similar to ATP binding in protein kinases. The N- and C-lobes are linked through a loop, which is termed the ‘hinge region’. The backbone of this loop interacts with the adenine moiety of ATP by hydrogen bonds. Considering the experimental result that piceatannol is an ATP-competitive inhibitor of PI3K (46), we docked the compound to the ATP binding site of PI3K (Fig. 5B). The B ring moiety of piceatannol partially overlapped and was coplanar with the space occupied by the adenine moiety of ATP, forming hydrogen bonds with the backbone of the hinge region of PI3K as other ATP-competitive PI3K inhibitors do (44). The hydroxyl group at the 5′ position acts as a hydrogen bonding acceptor in the interaction with the backbone amide group of Val882 and the hydroxyl group at the 4′ position is a hydrogen-bonding donor in the interaction with the backbone carbonyl group of Val882. The A ring moiety of piceatannol interacts with the N-lobe through hydrogen bonds. The hydroxyl group at the 3′ position can form a hydrogen bond with the side chain of Lys833, which interacts with the α-phosphate of ATP in the structure of PI3K in complex with ATP (45). In addition, the hydroxyl group at the 5′ position can form hydrogen bonds with the side chains of Asp841. The higher PI3K inhibitory activity of piceatannol over resveratrol can also be explained by the chemical structures of the two compounds. Based on previous competition studies with ATP, resveratrol also acts as a competitive inhibitor targeting the ATP-binding pocket (47). Therefore, the binding mode of resveratrol to PI3K would be similar to that of piceatannol due to the structural similarity between the two compounds. Piceatannol differs from resveratrol only by the addition of a hydroxyl group at the 5′ position in the B ring moiety. As a result of the additional hydrogen bond between the hydroxyl group and the backbone amide group of Val882, piceatannol is a more tight-binding inhibitor than resveratrol. Previously, we have reported that piceatannol inhibits PI3K activity in atherosclerotic models (46). In this study, using computer modeling tools, we further elucidated the structural evidence of piceatannol functioning as a PI3K inhibitor and clarified the differential effects of piceatannol and resveratrol based on their chemical structures.

In conclusion, piceatannol inhibits H-ras-induced MMP-2 expression, invasion, migration and anchorage-independent growth, whereas resveratrol has relatively minor inhibitory effects in MCF10A human breast epithelial cells. These inhibitory effects were mediated mainly through the blocking of the PI3K/Akt pathway. Piceatannol markedly suppressed PI3K activity by directly binding to PI3K. Our computer modeling data suggest that piceatannol is a more tight-binding inhibitor than resveratrol due to the additional hydrogen bond between the hydroxyl group and the backbone amide group of Val882 in the ATP binding pocket of PI3K.

## Abbreviations

ECM: extracellular matrix
ERK: extracellular signal-regulated kinase
MMPs: matrix metalloproteinases
NF-κB: nuclear factor-κB
PI3K: phosphatidylinositol 3-kinase
PIP_3_: phosphatidylinositol (3,4,5)-trisphosphate
PIP_2_: phosphatidylinositol (4,5)-bisphosphate
PDK: phosphoinositide-dependent kinase
H-ras MCF10A cells: MCF10A human breast epithelial cells harboring mutated H-ras
